# Spontaneous Adductor Hematoma in a 75-Year-Old Male With Acute Thigh Bruises

**DOI:** 10.7759/cureus.54079

**Published:** 2024-02-12

**Authors:** James Kim, Hye Chang Rhim, Jeffrey C Schneider

**Affiliations:** 1 Department of Physical Medicine and Rehabilitation, Boston University School of Medicine, Boston, USA; 2 Department of Physical Medicine and Rehabilitation, Spaulding Rehabilitation Hospital, Harvard Medical School, Boston, USA

**Keywords:** asymptomatic anemia, acute inpatient rehabilitation, multidisciplinary decision-making, intramuscular hematoma, point-of-care-ultrasound, diagnostic musculoskeletal ultrasound, complications of anticoagulation

## Abstract

Patients with a history of blood clots are commonly placed on anticoagulation therapy, but a common adverse effect of this therapy is hemorrhage. Our patient with a history of left ventricle thrombus in a free-standing inpatient rehabilitation hospital had ecchymoses that expanded over a large portion of the posterior thigh in one day. The rapid expansion of the ecchymoses coupled with a decline in hemoglobin levels prompted an immediate evaluation that showed an intramuscular hematoma. With hemoglobin levels dropping significantly, the patient was then transferred to the emergency department for higher levels of care. A multidisciplinary team of vascular surgery and electrophysiology physicians decided to discontinue anticoagulation and monitor the hematoma with serial compartment checks. The hematoma had stabilized throughout the hospital stay without surgical intervention. This case will discuss the difficulty of managing a patient with a history of previous blood clots with an adductor magnus hematoma in the setting of concurrent iatrogenic anticoagulation. Furthermore, we will review the current management and evaluation of intramuscular hematomas.

## Introduction

Major bleeding from antithrombotic agents is one of the most severe and common adverse effects, with a greater than six percent chance of a major bleeding incident occurring within five years of initiating treatment [1]. Gastrointestinal bleeding, intracranial hemorrhage, and intramuscular hematomas (IH) are common bleeding sites in anticoagulated patients [2,3]. The current literature on the epidemiology of IH due to oral anticoagulants is limited, but one population-based cohort study found 5.8% of patients with major bleeding related to oral anticoagulants to be major muscular hematomas [4]. The most feared complications of IH include compartment syndrome, major blood loss, and ischemia [4-6]. Risk factors for IH include microtrauma, iatrogenic causes from surgery, chronic renal insufficiency, and coagulopathy [7]. There are also case studies reported for anticoagulated patients who had an IH due to injections and muscular biopsies as rarer causes for IH [6,8-11]. However, many causes of IH have an unknown etiology.

## Case presentation

**Figure 1:**
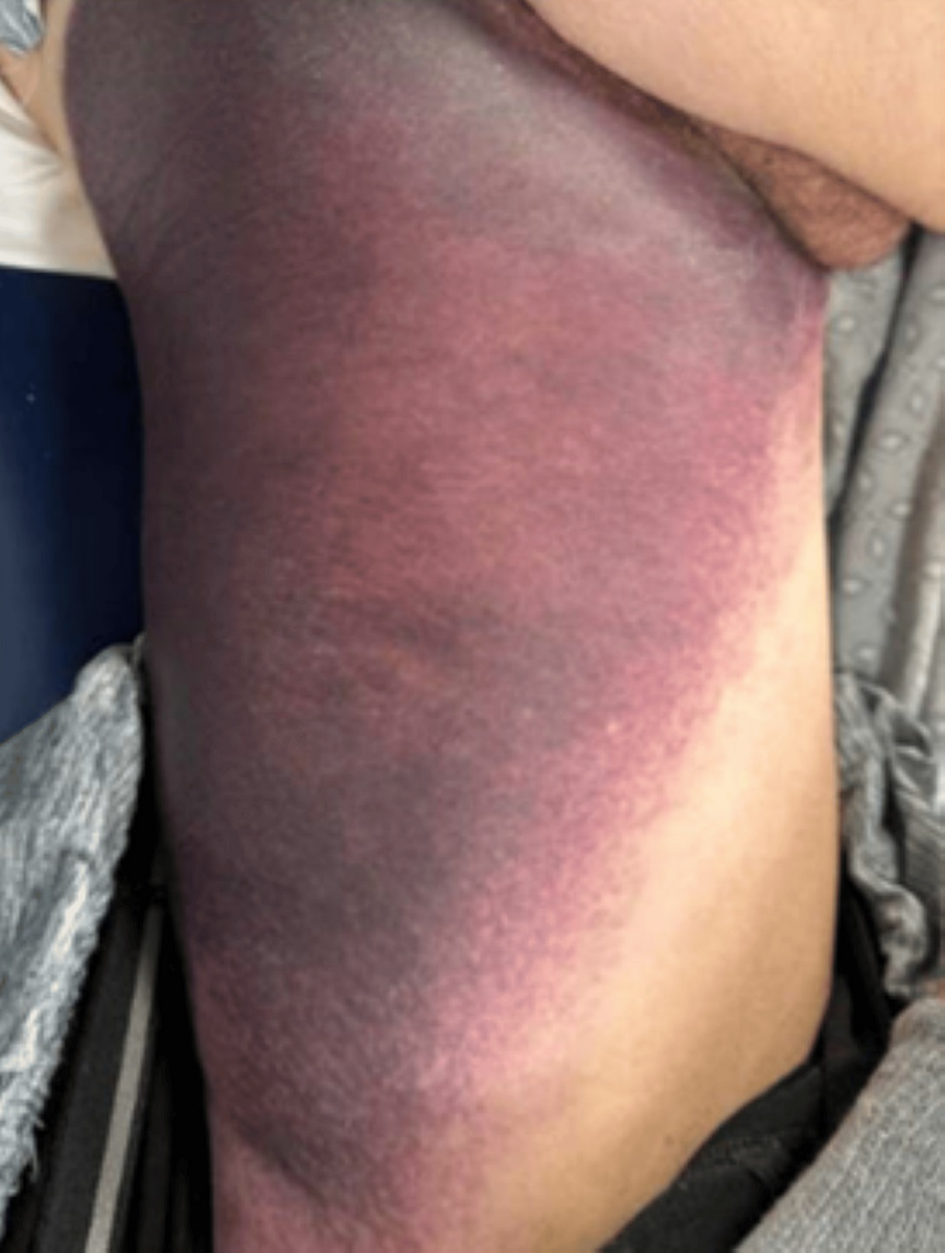
Large spontaneous posterior medial hematoma of the left thigh.

A 75-year-old male with a history of left ventricle thrombus on warfarin, heart failure with mid-range ejection fraction, hypertension, chronic kidney disease, and coronary artery disease was admitted to a free-standing inpatient rehabilitation hospital after a cardiac ablation performed 13 days prior for monomorphic ventricular tachycardia. The patient was started on 90 mg BID enoxaparin therapy bridging to warfarin in the acute hospital setting and continued at the rehabilitation facility. On rehabilitation day two, a small area of ecchymosis that developed without trauma in his posterior thigh was noted in the morning. The international normalized ratio (INR) level was 3.8, compared to 2.0 the day before, and the goal range was 2.0-3.0. Therefore, warfarin was held, and INR levels were monitored daily. Sequential compression devices were utilized as deep venous thrombosis (DVT) prophylaxis. By evening, the ecchymosis had expanded to a significant portion of the thigh (Figure 1). A point-of-care ultrasound (POCUS) demonstrated findings consistent with intramuscular hematoma. Hemoglobin level decreased from 11.2 mg/dL the day prior to 8.5 mg/dL in the morning and 7.6 mg/dL in the evening. However, the patient denied any shortness of breath, chest pain, dizziness, or lightheadedness, despite these concerning lab findings. Furthermore, the patient was afebrile and hemodynamically stable. Due to the patient's cardiac history, the hemoglobin threshold for acute care management was less than 8.0 mg/dL, and he was subsequently transferred to a tertiary care center for further evaluation.

**Figure 2:**
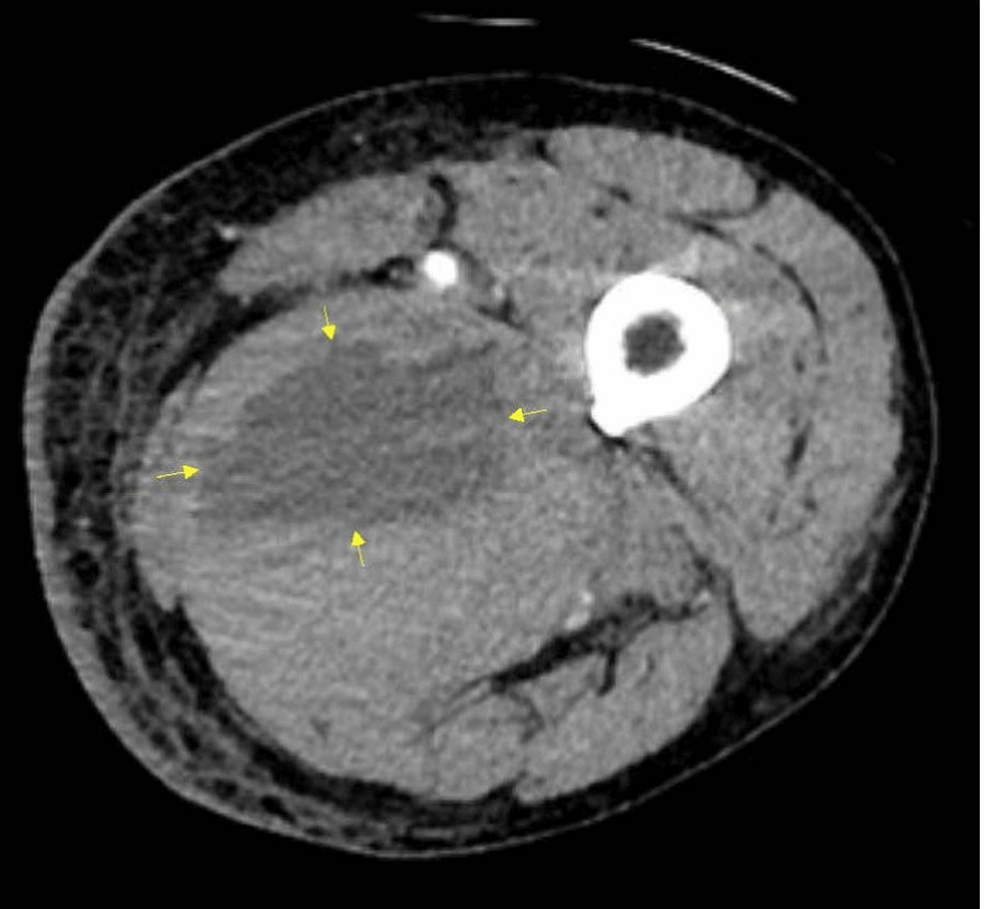
CT angiogram demonstrating hematoma within the adductor muscle.

**Figure 3:**
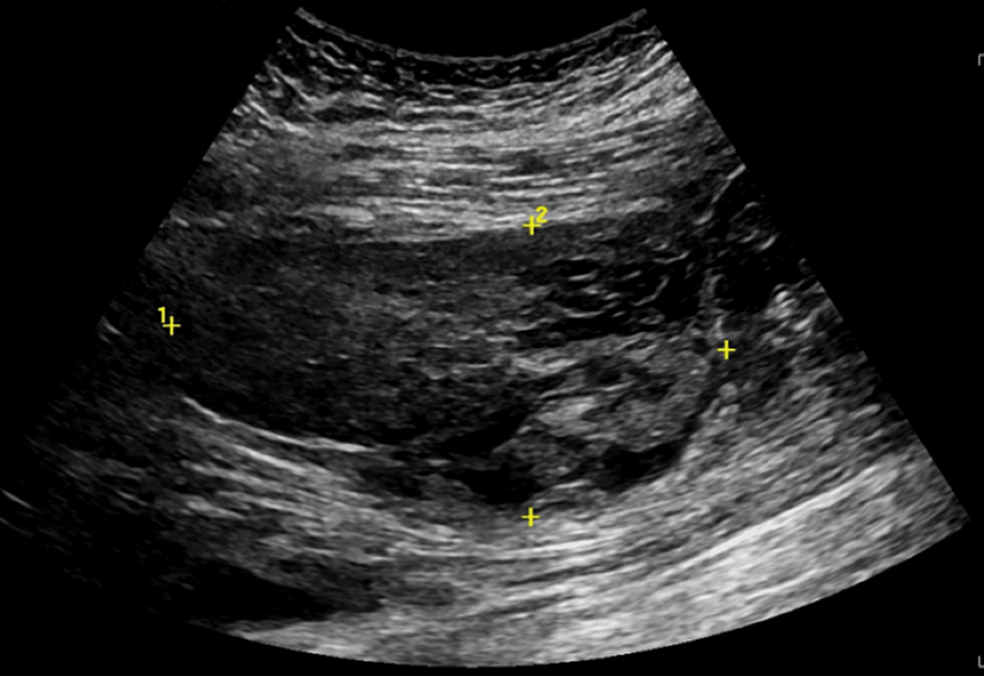
Ultrasound findings of a large hypoechogenic mass within muscle tissue with no Doppler flow are most consistent with a hematoma formation.

Upon being transferred, the patient was afebrile and hemodynamically stable. Physical exam findings showed intact sensation of bilateral lower extremities and 4/5 strength bilateral hip flexion with no other motor deficits. The patient had mild pain with hip internal rotation, mild tenderness to palpation of the medial-posterior thigh, full range of motion of the hip, and a negative FABER test. The skin was warm and dry, and the femoral and dorsalis pedis pulses were intact bilaterally. All findings were unchanged since the patient's admission to the rehabilitation hospital. The patient was emergently sent for a CT angiogram, which showed a pseudoaneurysm of the left femoral artery and multiple intramuscular hematomas in the adductors without evidence of active extravasation. The largest hematoma was found in the adductor magnus, measuring 6.8 x 4.0 x 7.1 cm (Figure 2). Ultrasound confirmed a large hypoechogenic mass within muscle tissue with no Doppler flow, most consistent with a hematoma formation measuring 10.0 x 5.2 x 9.6 cm in the left medial proximal thigh (Figure 3). Given the patient was hemodynamically stable and not actively bleeding, arterial embolization was deferred.

Vascular surgery and electrophysiology were consulted, and they recommended serial left groin ultrasounds and compartment and neurovascular checks. Both teams also agreed to withhold anticoagulation despite the thromboembolic risk due to concerning hemoglobin levels. On hospital day two, the patient received one unit of packed red blood cells (pRBCs) due to non-rising hemoglobin levels. The hemoglobin levels appropriately increased to 8.9 g/dL. However, hemoglobin levels the following day showed a decline to 7.1 g/dL, and the patient was administered another unit of pRBCs. A serial left groin ultrasound at that time showed no active bleeding in the left femoral artery, and the hemoglobin stabilized. The patient was restarted on 4 mg warfarin on hospital day three, and INR levels were monitored daily with the INR goal between 2 and 3. The patient's left groin pain improved, and he was discharged on hospital day eight back to the rehabilitation hospital. A follow-up appointment was made one month later with a vascular surgeon. 

## Discussion

Intramuscular hematomas are not an uncommon complication in the setting of anticoagulation (AC) therapy [12]. Risk factors for IH in anticoagulated patients include microtrauma, coagulation disorders, chronic renal insufficiency requiring dialysis, hypertension, heart failure, liver failure, and congenital collagen disorders [13]. Our patient, his history of chronic kidney disease, hypertension, and heart failure increases the risk for IH. A previous case series reported nine cases of incidences of IH in anticoagulated patients, but the quickly developing and fast-growing nature of this patient’s hematoma in less than 24 hours at the rehabilitation hospital was concerning [14]. Despite an active bleed and a hemoglobin level of 7.6 mg/dL, the patient was otherwise asymptomatic except for mild discomfort.

An asymptomatic patient with rapidly declining hemoglobin and expanding hematoma is an atypical presentation of an IH and emphasizes the need for thorough assessment in cases with mild signs or symptoms in the setting of bleeding risks such as supratherapeutic INR [14]. A free-standing rehabilitation hospital may lack the in-house specialists and imaging modalities necessary to monitor for hematomas, further suggesting the need for close observation, serial compartment examinations, and awareness of this diagnosis on the differential for anticoagulated patients with rapidly progressing anemia [15]. A diagnosis of IH is imperative due to the potential risk of sudden voluminous blood loss, ischemia, and compartment syndrome [4,14].

The evaluation for a rapidly expanding hematoma involves a thorough symptomatic review of pain and neurovascular dysfunction such as numbness, pallor, weakness, and hemodynamic instability such as dizziness or dyspnea, to which our patient denied all symptoms of anemia. Secondly, a thorough physical exam checking for paresthesia, swelling, weakness, and vital signs indicative of hemodynamic instability or compartment syndrome are vital to making an accurate diagnosis, which our patient fortunately had no signs of. Hemoglobin and prothrombin time (PT)/partial thromboplastin time (PTT)/INR levels must also be checked as evidence of active bleeding in the initial evaluation to search for a potential etiology [13].

Imaging modalities include a POCUS and CT angiogram with contrast. POCUS remains the initial diagnostic imaging of choice due to its availability at the bedside in most facilities, real-time imaging, and low cost [15]. The CT angiogram is relatively sensitive and specific (80 and 67 percent, respectively) [12]. An arteriogram is useful if surgical intervention is considered for active bleeding. In a care facility where imaging modalities are limited, a POCUS provides invaluable information. For this patient at a rehabilitation facility, ultrasound was vital in making the correct diagnosis. Furthermore, the ultrasound Doppler flow is essential when evaluating for active bleeding in real-time, which is difficult to assess with CT or MRI. Findings of a hypoechoic mass on the initial POCUS for this patient were crucial while evaluating for potential bleeding, further highlighting the value of ultrasound.

Management of a large intramuscular hematoma involves a clinical risk-benefit decision based on discontinuation of anticoagulation with the risk of thrombus formation [13]. A multidisciplinary decision is encouraged on anticoagulation therapy dosage and management. This decision is dependent on hemodynamic stability, hemoglobin level, and imaging findings determining the severity of blood loss. Furthermore, other complications, such as compartment syndrome, must also be monitored with serial physical examinations. In the case of neurovascular compromise due to compartment syndrome, an emergency fasciotomy with debridement should be considered for treatment [16]. However, image-guided aspirations could be considered as an alternative therapy to relieve the compression [15].

For hemodynamically unstable patients with declining hemoglobin levels, interventional radiology or surgery may be indicated in the setting of active bleeding confirmed by angiography. However, in a retrospective study of 73 patients with IH in the ED, 9.5% required arterial embolization. Most cases of hematomas had resolved spontaneously after reversal of AC therapy, and two patients experienced venous thromboembolic events [3]. This patient remained asymptomatic despite his hemoglobin dropping 3.6 mg/dL in a 24-hour period; a multidisciplinary consensus between vascular surgery and electrophysiology was achieved to hold anticoagulation. The patient was continuously monitored in the observation unit at an acute care facility, and his hemoglobin level adequately improved after receiving two units of packed red blood cell transfusion.

## Conclusions

Intramuscular hematomas are a potential adverse effect in patients on anticoagulation therapy. Initial evaluation includes precise history taking, a thorough physical exam, and imaging as required. In our patient with a history of thrombotic events, the clinical risks and benefits complicate the management of a bleeding patient. Without guidelines, much of the decision should be made from a multidisciplinary team that can provide acute levels of care. The complexity of the objective values and medical history of our patient adds difficulty to making clinical decisions about anticoagulation therapy. The risk of continuous bleeding while on anticoagulation versus a possible thrombotic event without it calls for interdisciplinary shared decision-making. Fortunately, the patient did not require any interventions besides discontinuing the anticoagulation.
